# Genotoxicity of Water Extract from Bark-Removed *Rhus verniciflua* Stokes

**DOI:** 10.3390/molecules26040896

**Published:** 2021-02-08

**Authors:** Sung-Bae Lee, Jin-Seok Lee, Jing-Hua Wang, Min-Young Kim, Yung-Hyun Choi, Hwa-Dong Lee, Chang-Gue Son

**Affiliations:** 1 Institute of Bioscience & Integrative Medicine, Daejeon University, 176 split 75 Daedeokdae-ro Seo-gu, Daejeon 35235, Korea; sky161300@naver.com (S.-B.L.); neptune@dju.kr (J.-S.L.); ewccwang@gmail.com (J.-H.W.); 2 Department of Biochemistry, Dong-eui University College of Korean Medicine, Busan 47227, Korea; ilytoo365@deu.ac.kr (M.-Y.K.); choiyh@deu.ac.kr (Y.-H.C.); 3 National Institute for Korean Medicine Development, Gyeongsan-si 38540, Korea; herb@nikom.or.kr

**Keywords:** genotoxicity, rhus verniciflua stokes, safety, reverse mutation, chromosomal aberration

## Abstract

*Rhus verniciflua Stokes* (RVS) has been traditionally used as an herbal remedy to support the digestive functions in traditional Korean medicine. Additionally, the pharmacological effects of RVS, including antioxidative, antimicrobial and anticancer activities, have been well-reported. The genotoxicity of RVS, however, is elusive; thus, we evaluated the genotoxicity of RVS without bark (RVX) for safe application as a resource of functional food or a medical drug. To evaluate the genotoxicity of RVX, we used a bacterial reverse mutation test, chromosomal aberration test and comet assay, according to the “Organization for Economic Co-operation and Development” (OECD) guidelines. Briefly, for the reverse mutation test, samples (5000, 1667, 556, 185, 62 and 0 μg/plate of RVX or the positive control) were treated with a precultured strain (TA98, TA100, TA1535, TA1537 or WP2µvrA) with or without the S9 mix, in which RVX partially induced a reverse mutation in four bacterial strains. From the chromosomal aberration test and comet assay, the RVX samples (556, 185, 62, 20 and 0 μg/mL of RVX or the positive control) were treated in a Chinese hamster ovary cell line (CHO-K1 cells) in the conditions of the S9 mix absent or S9 mix present and in Chang liver cells and C2C12 myoblasts, respectively. No chromosomal aberrations in CHO-K1 or DNA damage in Chang liver cells and C2C12 myoblasts was observed. In conclusion, our results suggest the non-genotoxicity of RVX, which would be helpful as a reference for the safe application of bark-removed *Rhus verniciflua Stokes* as functional raw materials in the food, cosmetics or pharmaceutical fields.

## 1. Introduction

Medicinal plants have been used in traditional medicine to treat various health problems from ancient times [1]. According to “Report Ocean”, the herbal products world market was estimated at 120 billion USD in 2019; this amount will grow up to 200 billion USD in 2024 [2]. In general, herbal products have been perceived by the public as relatively low risk. However, the continuous use of herbal products raised some concerns for the potential risks of these substances [3]. One review reported that 19 of 50 herbs had moderate or severe adverse effects [4]. Especially, hepatotoxicity or renal toxicity frequently occur as toxic effects caused by medicinal herbs [5,6]. These potential risks can occur by contamination, adulteration, the misidentification of herbs and interaction with other herbs, as well as the inherent toxicity of herbs; therefore, safety evaluations and the quality management of herbal products should be necessary for safe use [7].

The genotoxicity of herbal products sometimes becomes a potential risk in health [8,9]. In genetics, genotoxicity encompasses the property of chemical agents that damages the genetic information, causing mutations that may lead to cancer. The genotoxic substances can affect indirectly or directly damaged DNA, and these genetic properties can be passed to the next generation [9,10]. In several clinical studies, it has been reported that herbal products with genotoxicity could be risky, especially for pregnant women [11,12]. Thus, the “Korean Ministry of Food and Drug Safety” (MFDS) has recently announced a guideline (guidebook no. 2017-0290-02) that requires evaluation of the genotoxicity of new or modified herbal medicinal formulae [13]. 

*Rhus verniciflua* Stokes (RVS) has been traditionally used to treat mainly gastrointestinal disorders [14,15]. Several fractions and allergen-removed extracts from RVS have proven pharmacological effects, such as antioxidant [16,17], antimicrobial [18,19] or anti-inflammatory effects [20,21]. In addition, anticancer activities of RVS in allergen-removed extracts have been reported in both experimental [22,23,24] and clinical studies [25]. We also reported the antiemetic and myelo-protective effects of allergen-removed RVS extracts using a cisplatin-injected animal model [26]. 

In fact, RVS contains urushiol, a typical allergenic compound, mainly in the bark [27], which limits the medicinal application of this plant [28]. Several strategies to remove urushiol, such as high-temperature treatment, fermentation and fractionation, are being developed for application in herbal medicines [29,30,31]. The allergen-free RVS has been evaluated for safety and efficacy in animal and clinical studies [32,33,34]. The urushiol-free RVS is also expected to be used for foods and cosmetics [35]. However, the genotoxicity of RVS has not been examined to date. In the present study, we conducted two in vitro genotoxicity tests, a bacterial reverse mutation and mammalian chromosomal aberration test, on the water extract of RVS without bark (RVX).

## 2. Results

### 2.1. Fingerprint Analysis of RVX

**Figure 1:**
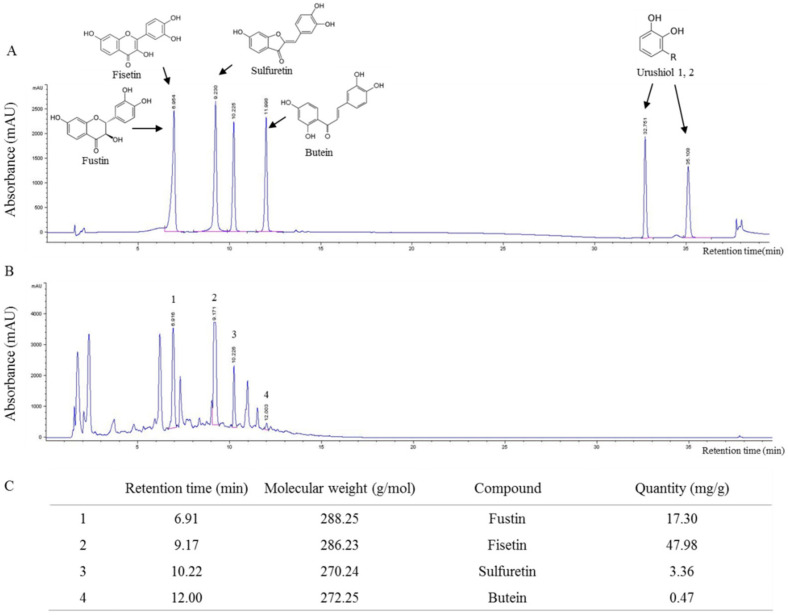
Fingerprint analysis: fingerprint analysis of four (**A**) standard compounds and (**B**) *Rhus verniciflua Stokes* without bark (RVX) using ultra-high-performance liquid chromatography-tandem mass spectrometry (UHPLC-MS/MS). (**C**) Quantitative analysis of RVX was performed by ChemStation software.

From the analysis using ultra-high-performance liquid chromatography-tandem mass spectrometry (UHPLC-MS/MS), four main compounds of RVX were identified at retention times of 6.91, 9.17, 10.22 and 12.00 min for fustin, fisetin, sulfuretin and butein, respectively (Figure 1A,B). Their molecular weights were confirmed as follows: fustin, 288.25 g/mol, fisetin, 286.23 g/mol, sulfuretin, 270.24 g/mol and butein, 272.25 g/mol. We also obtained semi-quantitative data for them: fustin, 17.3 mg/g, fisetin, 47.98 mg/g, sulfuretin, 3.36 mg/g and butein, 0.47 mg/g (Figure 1C). Both urushiol 1 and urushiol 2 were not detected in RVX (Figure 1A,B).

### 2.2. Determination of Reverse Mutation in Bacterium

**Figure 2:**
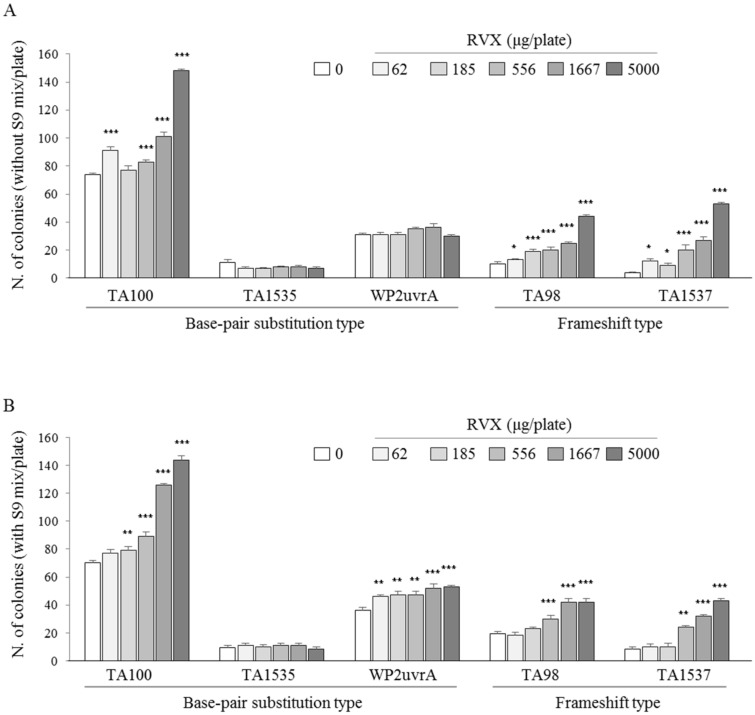
Bacterial reverse mutation test was performed according to the Organization for Economic Co-operation and Development (OECD) guideline number 471. The RVX or positive control was treated for each bacterial strain under the conditions of the S9 mix: (**A**) absence or (**B**) presence of the mix after incubation. The revertant colonies were counted, and the above test was repeated in triplicate. * *p* < 0.05, ** *p* < 0.01 and *** *p* < 0.001 compared to the negative control (0 μg/mL).

The RVX (62, 185, 556, 1667 and 5000 μg/plate) were treated for five bacterial strains (TA100, TA1535, WP2uvrA, TA98 and TA1537) under the absence or presence of the S9 mix. Regardless of whether the S9 mix was present, the cytotoxicity of RVX not observed in all strains. Under the absence of S9 mix, the RVX treatment significantly increased the number of colonies in the TA100 strain (from 62 μg/plate, except 185 μg/plat, *p* < 0.001) and TA98 and TA1537 strains (from 62 μg/plate, *p* < 0.05 or *p* < 0.001) compared to the negative control (Figure 2A). Under the presence of the S9 mix, RVX-related reverse mutations were observed in four strains: TA100 (from 185 μg/plate, *p* < 0.01 or *p* < 0.001), WP2uvrA (from 62 μg/plate, *p* < 0.01 or *p* < 0.001), TA98 (from 556 μg/plate, *p* < 0.001) and TA1537 (from 556 μg/plate, *p* < 0.01 or *p* < 0.001), respectively (Figure 2B). As expected, all positive control agents (AF2, NaN3, 4-NQO and 9-AA for the condition without the S9 mix and 2-AA and BP for the condition with the 9S mix) showed a significant reverse mutation at least three-fold (*p* < 0.001; Table 1).

### 2.3. Determination of Chromosomal Aberration in a Hamster Ovary Cell Line

**Figure 3:**
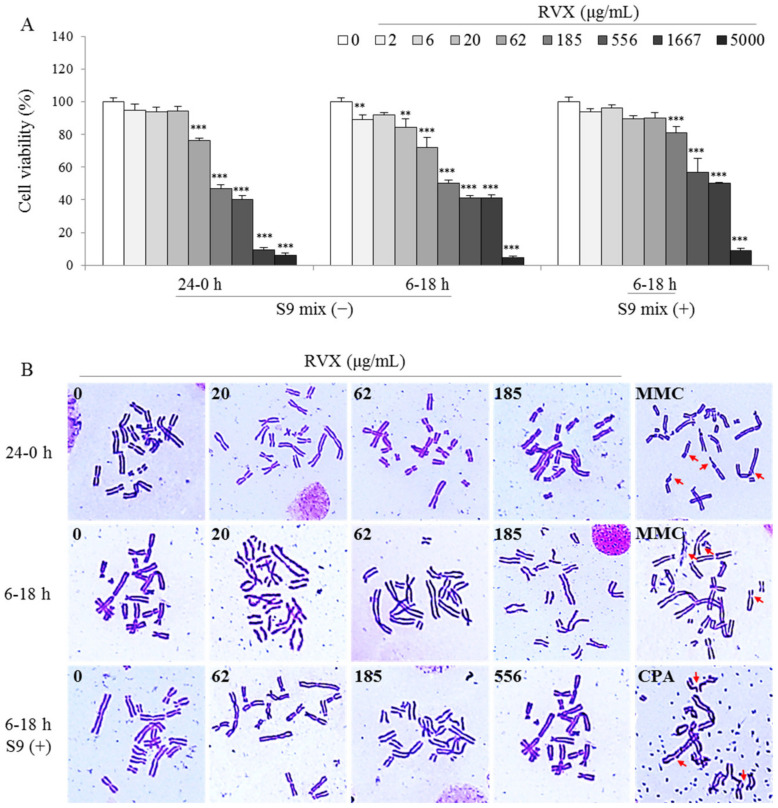
(**A**) Chinese hamster ovary cell line (CHO-K1) cell viability was determined using a 3-(4,5-dimethylthiazol-2-yl)-2,5-diphenyltetra-zolium bromide (MTT) assay. (**B**) Chromosomal aberration CHO-K1 cells were treated RVX or MMC for 24 h for long-term treatment, while cells were treated with RVX or MMC for 6 h, followed by incubation in fresh media for a further 18 h, under the two conditions (absence or presence of the S9 mix) for the short-term treatment. The structural and numerical chromosomal aberrations were counted under an optical microscope. ** *p* < 0.01 and *** *p* < 0.001 compared to the negative control (0 μg/mL). MMC, Mitomycin C.

The RVX (2–5000 μg/mL) were treated to Chinese hamster ovary cell line (CHO-k1) cells under the absence or presence of the S9 mix for a short (18-h recovery after 6-h incubation) or long time (24-h incubation), and the half-maximal inhibitory concentrations (IC_50_) were calculated as 191 and 489 μg/mL in the presence and absence of the S9 mix via the cell viability assay, respectively (Figure 3A). The chromosomal aberration test was performed based on the IC_50_ (maximum 185 and 556 μg/mL in the presence and absence of the S9 mix, respectively). Structural or numerical aberrations of the RVX treatment were not observed, regardless of the presence of the S9 mix (Figure 3B), and the number of chromosomal aberrations were counted (*p* > 0.05; Table 2). As expectation, the positive chemicals (mitomycin C and cyclophosphamide) significantly induced the chromosomal aberrations (*p* < 0.001; Figure 3B and Table 2).

### 2.4. Determination of DNA Damage in Human and Mouse Cell Lines

**Figure 4:**
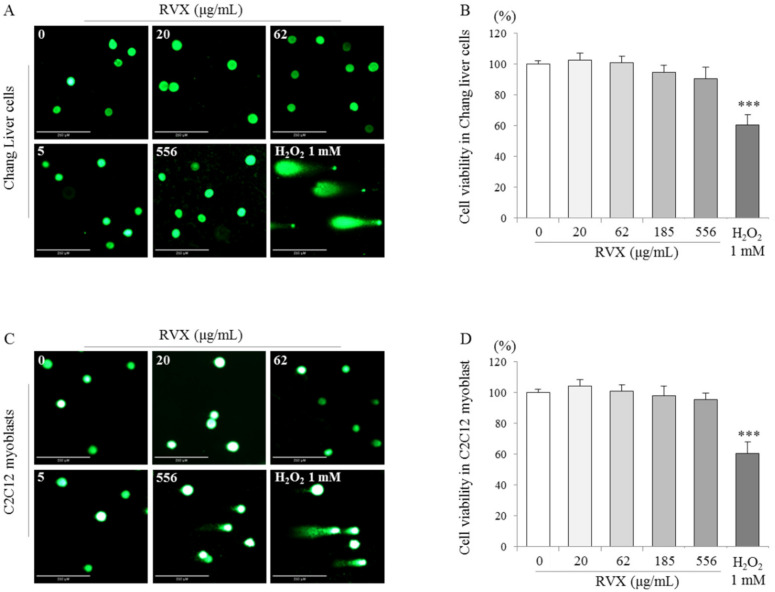
(**A**,**C**) A comet assay was performed for Chang liver and C2C12 cells after treatment with RVX for 24 h. The images stained with propidium iodide (PI) were captured under a fluorescence microscope. (**B**,**D**) The viability of Chang liver and C2C12 cells was determined using the MTT assay. *** *p* < 0.001 compared to the negative control (0 μg/mL).

To exclude DNA damage by the cytotoxicity of RVX, a cell viability assay was performed at a 20–556 μg/mL concentration of RVX in Chang liver and C2C12 cells. The treatment of RVX did not induce cytotoxicity in the two types of cells (Figure 4B,D). Based on the cell viability results, RVX or 1 mM of H_2_O_2_ was treated to Chang liver and C2C12 cells. Compared to nontreated cells, no differences of DNA tail length by RVX treatment were observed in both Chang liver and C2C12 cells (Figure 4A,C), while the treatment of H_2_O_2_ dramatically increased the DNA tail length compared to nontreated cells in two types of cells (Figure 4A,C).

## 3. Discussion

Genotoxic substances can lead to malformations or carcinogenesis; therefore, to assess the genotoxicity is a key factor for the protection of human health [36]. For the wide adaptation of RVS as a natural resource for medicinal or food products, we evaluated the genotoxicity of RVS. The genotoxicity of RVS was performed using a reverse mutation test, mammalian chromosomal aberration test and comet assay related to DNA damage, according to the OECD guidelines. In the present data, RVX induced reverse mutations of four bacteria, excluding TA1535, in a bacterial reverse mutation test but not chromosomal aberration in CHO-K1 cells and DNA damage in Chang liver and C2C12 cells.

The reverse mutation test has been used worldwide as an initial screen to determine the mutagenic potential of new chemicals and drugs. In our results, RVX induced mutagenesis on TA98, TA100, TA1537 and WP2µvrA in the conditions of the absence or presence of theS9 mix (Figure 2). These positive results indicated that the tested sample possibly causes a frame shift or point mutation, including the addition, deletion or substitution of one [37]. However, the genotoxic mechanism by which RVX induces these mutations is unclear.

To reverify the above results, we additionally performed chromosomal aberration in a hamster cell line and the comet assay in the human and mouse cell lines. The mammalian chromosomal aberration test was used to identify possible mutagens and carcinogens [38]. Chromosomal mutations cause various genetic diseases, and there is substantial evidence that chromosomal mutations are found in oncogenes and tumor-suppressor genes [39]. As we could expect, two positive chemicals, mitomycin C and cyclophosphamide, significantly induced both structural and numerical aberrations, regardless of the presence of the S9 mix (*p* < 0.001), while they were not observed in the RVX treatment group (Figure 3 and Table 2). In addition, RVX did not show an alteration of DNA in the human and mouse cell lines, according to the results from the comet assay (Figure 4). The comet assay is a versatile and simple technique used to measure DNA damage and repair in individual cells [40]. Oxidative DNA damage by reactive oxygen species (ROS) and free radicals are important in the pathogenesis various diseases, and H_2_O_2_, one of the main ROS, is known to cause oxidative DNA damage in various cells [41]. These negative results from both the chromosomal aberration test and comet assay may indicate the absence of possibility of RVX genotoxicity. Like the above descriptions, RVX showed conflicted results in three types of genotoxicity in vitro tests. It has frequently been reported that many noncarcinogens can produce false-positive results in certain genotoxicity assays [42]. If the mammalian chromosomal aberration test and comet assay showed negative outcomes, a positive result in the bacterial reverse mutation test could be denied [43,44,45].

The bark of RVS mainly contains urushiol, a typical allergenic compound [27], and limits the medicinal application of RVS [28]. As we expected, no urushiol was detected in the sample (RVX, bark-removed RVS) of the present work (Figure 1), and the data was consistent with another study regarding the quantitative analysis of urushiol from bark-removed RVS [46]. Urushiol-free RVS is used as an anticancer remedy in Korea [47,48]. Some herbs are known to have genotoxicity, and the major compounds responsible for genotoxicity are those of the pyrrolizidine alkaloid series [49]. In the UHPLC-MS/MS analysis, fustin, fisetin, sulfuretin and butein were detected as the main compounds in RVX (Figure 1). It is well-known that RVS does not contain pyrrolizidine alkaloid compounds [50]. In contrast to our results, one study reported that water and ethanol extracts from RVS have a genotoxicity [51]; however, the reason may be the presence or absence of a bark. Bioactive compounds from the bark of RVS were also studied by many researchers [27,31,52], but its genotoxic activity is yet unknown. Further studies, including a micronucleus test, would be helpful in verifying the genotoxic safety of bark-removed RVS in the next study.

Taken together, our results from three types of in vitro tests revealed the non-genotoxicity of RVS. Although the present study had limitations, such as no information from in vivo tests, our data would be helpful as a reference for the safe application of bark-removed *Rhus verniciflua Stokes* as a functional raw material in the food, cosmetic or pharmaceutical fields.

## 4. Materials and Methods

### 4.1. Preparation of RVS and Fingerprinting Analysis

Water extract of RVS without bark (RVX) was obtained from Daehan Bio Pharm Inc. (Gyeonggi-do, South Korea). Briefly, 100 kg of the bark, excluding the wooden part of RVS, was boiled in 1000 L of distilled water at 100 °C for 2 h. After centrifugation, the supernatant was filtered through Adventec filter paper (Toyo Roshi Kaisha, Tokyo, Japan). The filtered extract was lyophilized using an Ecospin 3180C (Hanil Science Medical, Daejeon, South Korea). Finally, RVX powder was obtained (final yield: 0.71%) and stored at −70 °C in a deep freezer for future use.

To identify the chemical components of RVX, fingerprinting analysis was conducted using an ultra-high-performance liquid chromatography-tandem mass spectrometry (UHPLC-MS/MS; Agilent Technologies, Santa Clara, CA, USA). Briefly, five milligrams of RVX were dissolved in 1 mL of 50% methanol, and the solution was filtered. Sample solutions of 10 μL were subjected to UHPLC-MS/MS using an MS Spectrometer (Thermo Fisher Scientific, Santa Clara, MA, USA). Separation was performed using the C18 column (4.6 nm × 150 nm and particle size, 5 μm; Waters, Torrance, CA, USA) at 50 °C. The column was eluted at a flow rate of 0.35 mL/min using water (in 0.1% formic acid) and acetonitrile (in 0.1% formic acid), which were used as mobile phases A and B, respectively. The following gradients were applied: 0 to 1 min, 0–1% B in A, 1–7 min, 1–100% B in A and 7–10 min, 100–1% B in A (linear gradient). The compositional analyses were conducted using a photodiode array at 200–600 nm. The full-scan mass spectra were acquired at 100–1000 *m*/*z* in positive and negative modes. The data were acquired by ChemStation software (Agilent Technologies, Wilmington, DE, USA) as compared to the five reference compounds (fustin, fisetin, sulfuretin, urushiol 1 and urushiol 2). Quantitative analysis of the major compounds in RVX was performed using UHPLC-MS/MS.

### 4.2. Bacterial Reverse Mutation Test

For the bacterial reverse mutation test, 4 histidine-requiring *Salmonella typhimurium* strains (TA98, TA100, TA1535 and TA1537) and one tryptophan-requiring *Escherichia coli* strain (WP2µvrA) were obtained from Molecular Toxicology (Boone, NC, USA). A minimal glucose agar plate (Junsei Chemical, Tokyo, Japan), D-( + )-glucose and Vogel-Bonner medium E (10×, Sigma Aldrich, St. Louis, MO, USA) were used. The top agar was combined with NaCl and Bacto agar (BD Korea, Busan, Republic Korea) with 0.5-mM histidine/biotin or 0.5-mM tryptophan (Sigma Aldrich, St. Louis, MO, USA). As the positive control, sodium azide (NaN_3_), 9-aminoacridine (9-AA), 2-(2-furyl)-3-(5-nitro-2-furyl) acrylamide (AF-2, 0.01 or 0.1 μg/plate), 2-aminoanthracene (2-AA), benzo(a)pyrene (BP) and 4-nitroquinoline 1-oxide (4-NQO) were obtained from Sigma Aldrich (St. Louis, MO, USA) or Wako Chemicals (Richmond, VA, USA).

The tests were performed by Korean Conformity Laboratory (Incheon, Republic of Korea, Test # GT17-00313), according to Organization for Economic Co-operation and Development (OECD) guideline number 471 (adapted on 21 July 1997) and a previous study [53]. The RVX were treated for each bacterial strain under the conditions with or without the S9 mix. Briefly, 0.05 mL of serum-free media; RVX (5000, 1667, 556, 185 and 62 μg/plate) or positive control (AF-2 0.01 or 0.1 μg/plate; NaN_3_ 0.5 μg/plate; 4-NQO 0.25 μg/plate; 9-AA 80 μg/plate; 2-AA 1.0, 2.0 or 10 μg/plate or BP 1.0, 2.0 or 10 μg/plate) were mixed with 2.0 mL of top agar and 0.1 mL of the precultured strain (TA98, TA100, TA1535, TA1537 or WP2µvrA) with 0.5 mL of the S9 mix or 0.1-M phosphate-buffered saline (PBS; pH 7.4). The mixture was vortexed and then poured onto a minimal glucose agar plate. After the agar overlay solidified, the plates were incubated for 48 h at 37 °C. After incubation, the revertant colonies were counted. The above test was repeated in triplicate.

### 4.3. Cell Cultures

CHO-K1 cell, a Chinese hamster ovary cell line, was purchased from the Korean Cell Line Bank (Seoul, Republic of Korea). Chang liver cells (human liver cell line) and C2C12 myoblasts (mouse muscle cell line) were obtained from the American Type Culture Collection (ATCC, Raymond, VA, USA). CHO-k1 and Chang liver cells were cultured in minimum essential medium (MEM; WelGENE Inc., Kyeong-book, Korea) supplemented with 10% fetal bovine serum (FBS; Thermo Fisher Scientific, Santa Clara, MA, USA) at 37 °C and 5% CO_2_. The C2C12 myoblast was cultured in Dulbecco’s modified Eagle’s medium (DMEM; WelGENE Inc., Kyeong-book, Korea) containing 10% FBS at 37 °C with 5% CO_2_.

### 4.4. Cell Viability Assay

The cell viability was determined by the ability of the mitochondria using conversion from 3-(4,5-dimethylthiazol-2-yl)-2,5-diphenyltetra-zolium bromides (MTT; Sigma-Aldrich, St. Louis, MO, USA) to formazan dyes. Briefly, CHO-K1, Chang liver and C2C12 cells were cultured in 96-well plates (1×10^4^ cells per well), respectively. After overnight, each cell was treated with various concentrations of RVX (from 2 to 5000 μg/mL) for 24 h. Following the incubation, the medium was removed, and the cells were supplemented with fresh medium containing the MTT solution (final concentration of 0.5 mg/mL) into each well and incubated for 3 h at 37 °C with 5% CO_2._ The MTT was removed, and cells were lysed with 150-μL Dimethyl Sulfoxide. Absorbance was measured at 570 nm using a microplate reader (VersaMax Molecular Devices, Sunnyvale, CA, USA). The optical density of formazan crystals formed in nontreated control cells was used to indicate 100% viability.

### 4.5. In Vitro Mammalian Chromosomal Aberration Test

The test was conducted by Korean Conformity Laboratory (Incheon, Republic of Korea, Test # GT17-00314) according to OECD guideline no. 473 (adapted on 29 July 2016) and a previous study [53]. Mitomycin C (MMC; Sigma Aldrich, St. Louis, MO, USA) or cyclophosphamide monohydrate (CPA; Abcam, Cambridge, UK) were used as positive control agents for the S9 mix absent or present conditions, respectively. Briefly, the CHO-k1 cells were incubated in 60-mm plates (4 × 10^4^ per plate) for 3 days at 37 °C and CO_2_. For the short-term treatment, cells were treated with RVX (185, 62 and 20 μg/mL) or MMC (0.04 μg/mL) for 6 h under the two condition (absence or presence of the S9 mix). After treatment, cells were washed and incubated in fresh media for a further 18 h. For the long-term treatment, cells were treated RVX (185, 62 and 20 μg/mL) or MMC (0.04 μg/mL) for 24 h. Colcemid (Thermo Fisher Scientific, Santa Clara, MA, USA) was added (10-μg/mL final concentration) at 2 h prior to harvesting. Harvested cells were added to 75-mM potassium chloride (KCL; Sigma Aldrich, St. Louis, MO, USA) and fixed in Carnoy’s solution (methanol:acetic acid, 1:3). Then, the cells were placed on glass slides and stained in 5% Giemsa (Merck, Kenilworth, NJ, USA). The structural and numerical chromosomal aberrations were counted under an optical microscope (Sinjuku, Tokyo, Japan).

### 4.6. Comet Assay in Human Liver and Mouse Muscle Cells

To determine deoxyribonucleic acid (DNA) damage by RVX, a comet assay was performed according to a previous study [54]. Briefly, the Chang liver and C2C12 cells were washed with PBS, suspended in low-melting agarose (LMA) at 37 °C and then spread on the microscope slides, which were precoated with normal melting agarose. After the agarose was solidified, the slides were covered with LMA and then submerged in lysis solution at 4 °C for 1 h. The slides were incubated in a gel electrophoresis device (Hercules, CA, USA) for 30 min and electrophoresed for 20 min at 30 V and 300 mA. Subsequently, the slides were washed with neutralizing buffer and then stained with propidium iodide (PI) (20 μg/mL, Sigma Aldrich, St. Louis, MO, USA). The images stained with PI were captured under a fluorescence microscope (Carl Zeiss, Oberkochen, Germany).

### 4.7. Statistical Analysis

Statistical analyses were performed for the bacterial reverse mutation test and in vitro chromosomal aberration test by one-way analysis of variance (ANOVA), followed by Tukey’s HSD (honest significant difference) post-hoc test using SPSS (IBM, San Francisco, CA, USA). In all analyses, *p* < 0.05, *p* < 0.01 or *p* < 0.001 was used as the threshold to indicate statistical significance. All data were expressed as the mean ± SD.

## Figures and Tables

**Table 1:** Positive agents for bacterial reverse mutations with or without S9 activation.

Metabolic Activation	Dose (μg/plate)	Number of Colonies/Plate				
		Base-Pair Substitution Type			Frameshift Type	
		TA100	TA1535	WP2uvrA	TA98	TA1537
S9 mix ( − )	Positive	AF-2	NaN_3_	4-NQO	AF-2	9-AA
	Dose	0.01	0.5	0.25	0.1	80
	Mean ± SD	545 ± 31.1 ***	281 ± 39.3 ***	318 ± 83.0 ***	499 ± 9.0 ***	1185 ± 146 ***
S9 mix ( + )	Positive	2-AA	2-AA	2-AA	BP	2-AA
	Dose	1.0	2.0	10.0	10.0	2.0
	Mean ± SD	309 ± 76.4 ***	193 ± 6.4 ***	263 ± 52.6 ***	522 ± 90.3 ***	143 ± 7.5 ***

Bacterial reverse mutation test was performed in four histidine-requiring *Salmonella typhimurium* strains: TA98, TA100, TA1535 and TA1537 and one tryptophan-requiring *Escherichia coli* strain, WP2µvrA. All data were expressed as the mean ± SD (*n* = 3). * *p* < 0.05, ** *p* < 0.01 or *** *p* < 0.001 compared to the negative control (0 μg/plate). NaN3, Sodium azide; 9-AA, 9-aminoacridine; AF-2, 2-(2-furyl)-3-(5-nitro-2-furyl) acrylamide; 2-AA, 2-aminoanthracene; BP, benzo(a)pyrene and 4-NQO, 4-nitroquinoline 1-oxide.

**Table 2:** Chromosomal aberrations by *Rhus verniciflua Stokes* without bark (RVX) with or without S9 activation.

Condition	Dose (μg/mL)	Aberration (No. in 300 cells)					Aberration Rate (%)
		Chromatid		Chromosome		PP+ER	
		Breaks	Exchange	Breaks	Exchange		
0–24 h S9 mix( − )	0	0	2	0	0	0	0.67
	20.58	1	0	0	0	0	0.33
	61.73	0	1	0	0	0	0.33
	185.19	2	1	0	0	0	1.00
	MMC 0.04	9	62	0	0	0	23.67 ***
6–18 h S9 mix( − )	0	1	0	0	0	0	0.33
	20.58	1	1	0	0	0	0.67
	61.73	1	1	0	0	0	0.67
	185.19	1	2	0	0	0	1.00
	MMC 0.04	12	54	0	0	0	22.00 ***
6–18 h S9 mix( + )	0	0	2	0	0	0	0.67
	61.73	0	1	0	0	0	0.33
	185.19	1	0	0	0	0	0.33
	555.56	1	2	0	0	0	1.00
	CPA 10	7	64	0	0	0	23.67 ***

Structural (break and exchange in chromatid or chromosome) and numerical aberrations (polyploidy and endoreduplication) were counted in 300 Chinese hamster ovary cell line (CHO-k1) cells. *** *p* < 0.001 compared to the negative control (0 μg/mL). MMC, Mitomycin C; CPA, Cyclophosphamide; PP, Polyploidy and ER, Endoreduplication.

## Data Availability

The data presented in this study are available on request from the corresponding author.

## Sample Availability

Samples of the Water extract of RVS without bark (RVX) are available from the authors.
